# Stabilizing Fe–N–C Catalysts as Model for Oxygen Reduction Reaction

**DOI:** 10.1002/advs.202102209

**Published:** 2021-10-23

**Authors:** Qianli Ma, Huihui Jin, Jiawei Zhu, Zilan Li, Hanwen Xu, Bingshuai Liu, Zhiwei Zhang, Jingjing Ma, Shichun Mu

**Affiliations:** ^1^ State Key Laboratory of Advanced Technology for Materials Synthesis and Processing Wuhan University of Technology Wuhan 430070 P. R. China; ^2^ Foshan Xianhu Laboratory of the Advanced Energy Science and Technology Guangdong Laboratory Xianhu Hydrogen Valley Foshan 528200 P. R. China

**Keywords:** electrocatalysis, fuel cells, oxygen reduction reaction, stability, TM–H–C electrocatalysts

## Abstract

The highly efficient energy conversion of the polymer‐electrolyte‐membrane fuel cell (PEMFC) is extremely limited by the sluggish oxygen reduction reaction (ORR) kinetics and poor electrochemical stability of catalysts. Hitherto, to replace costly Pt‐based catalysts, non‐noble‐metal ORR catalysts are developed, among which transition metal–heteroatoms–carbon (TM–H–C) materials present great potential for industrial applications due to their outstanding catalytic activity and low expense. However, their poor stability during testing in a two‐electrode system and their high complexity have become a big barrier for commercial applications. Thus, herein, to simplify the research, the typical Fe–N–C material with the relatively simple constitution and structure, is selected as a model catalyst for TM–H–C to explore and improve the stability of such a kind of catalysts. Then, different types of active sites (centers) and coordination in Fe–N–C are systematically summarized and discussed, and the possible attenuation mechanism and strategies are analyzed. Finally, some challenges faced by such catalysts and their prospects are proposed to shed some light on the future development trend of TM–H–C materials for advanced ORR catalysis.

## Graphical abstract

The poor stability is a huge limitation for the development of transition metal–heteroatoms–carbon (TM–H–C) as promising catalysts toward the oxygen reduction reaction. Accordingly, the stability of different active sites, possible deactivation mechanisms, and strategies to improve stability around classic Fe–N–C model are summarized and discussed, with an attempt to provide insight into reasonably constructing stable and efficient TM–H–C catalysts.

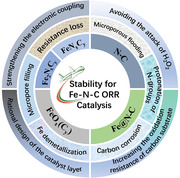

## 1 Introduction

With the consumption of fossil fuels, the energy shortage and serious pollution have become the major issues related to the sustainable development of human society. Thus, the development of renewable and clean energy has inevitably become the research focus. Among promising high‐efficiency electrochemcial energy conversion devices, polymer‐electrolyte‐membrane fuel cells (PEMFCs) have been considered the most ideal next‐generation energy conversion facility due to its nearly zero‐emission, wide application range, and high energy density.^[^ ^1^ ^]^ The energy conversion process of PEMFCs is mainly decided by the sluggish oxygen reduction reaction (ORR) kinetics owing to involving a multistep proton‐coupled electron transfer. In the oxygen reduction process, oxygen molecules are converted to H_2_O_2_ through a two‐electron pathway, or reduced to OH^−^ or H_2_O in an alkaline or acidic environment through a four‐electron pathway, respectively.^[^ ^2^ ^]^ In fact, most of the electrocatalysis reactions are a mixing process of two and four electron paths.^[^ ^3^ ^]^ Pt‐based materials are regarded as ideal catalysts for promoting the ORR process due to good catalytic performance, but high expense and scarcity of resources seriously limit their large‐scale applications.^[^ ^4^ ^]^ Therefore, developing cost‐efficient noble‐metal‐free ORR catalysts is urgent and of great significance.

In the past period of time, a large number of noble‐metal‐free ORR catalysts including transition metal oxides, phosphides, carbides, sulfides, and heteroatom‐doped carbon materials have been comprehensively studied.^[^ ^5^ ^]^ One of them, nitrogen‐doped carbon (N–C) materials with low cost, large specific surface area, and good electrocatalytic activity, have received wide attention.^[^ ^5^, ^6^ ^]^ Studies have shown that oxygen molecules are more likely to be activated on carbon atoms with more positive charges in C–N, this is because the nitrogen dopant with lone pairs of electrons can interact with the p electrons of sp^2^ C in carbon materials through electronic coupling effects.^[^ ^6^, ^7^ ^]^ Moreover, compared to C atoms, N atoms have one more electron and a similar radius, conducive to the ORR process that requires electrons.

In addition, incorporating atomically dispersed transition metal (TM) atoms (Fe, Mn, Ni, Cu, Co, etc.) and heteroatoms (H, such as N, S, P, B, etc.) into carbon frameworks to form TM–H–C structures can further boost the ORR performance of non‐noble‐metal catalysts.^[^ ^8^ ^]^ it is worth noting that such catalysts are readily suffered fast decay, especially in the actual working environment, which greatly restrains the commercial application of TM–H–C catalysts.^[^ ^9^ ^]^ Finally, multiple efforts have been devoted to synchronously achieving the TM–H–C catalyst with superior activity and durability/stability.^[^ ^10^ ^]^ However, in practical applications, for the catalyst, there is still a big gap in the performance compared to the commercial Pt‐based catalyst, especially in acidic conditions.^[^ ^11^ ^]^ The preparation of stable and efficient TM–H–C catalysts still faces many challenges. However, there are almost no reports concerning the improvement of the stability toward TM–H–C due to the complex constitution and structure. Among TM–H–C catalysts, due to the relatively simple constitution and structure, the Fe–N–C catalyst is the most classical, and considered to be easy to understand the catalysis mechanism of TM–H–C catalysts.^[^ ^12^ ^]^ Therefore, the rational design of Fe–N–C catalysts with improved stability is the sticking point to further obtain high‐performance TM–H–C non‐noble‐metal catalysts toward industrial applications.

**Scheme 1:**
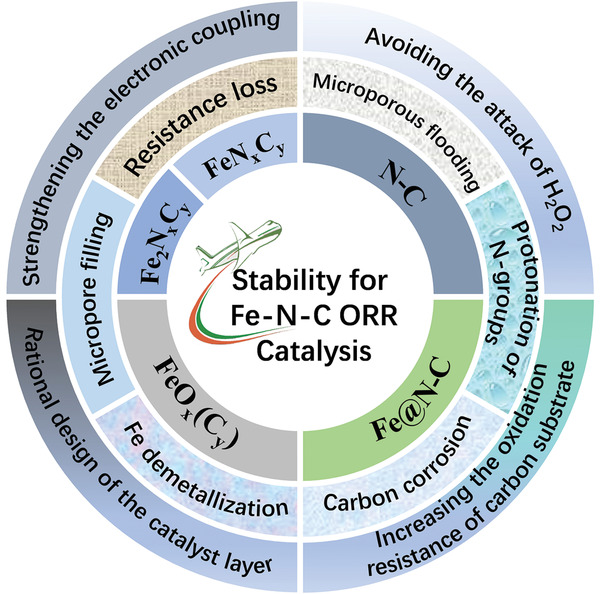
Typical Fe–N–C‐based catalysts for the oxygen reduction reaction.

Accordingly, herein, as illustrated in **Scheme** **1**, we summarize, analyze and discuss the relationship between active sites (centers) and stability of Fe–N–C catalysts, the main reasons for catalytic performance loss, and possible strategies to boost the stability of Fe–N–C catalysts. In view of the current understanding of Fe–N–C materials, key issues and feasibility prospects are proposed.

## 2 Catalytic Active Center and Coordination of Fe–N–C Catalysts

**Figure 1:**
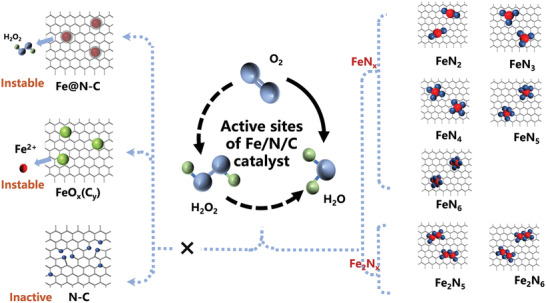
Active sites of Fe–N–C‐based catalysts for oxygen reduction reaction.

As a standard ORR catalyst, Fe–N–C has received extensive attention, however, the divergence in the understanding of active sites (centers) hinders the further development of such a catalyst. So far, as shown in **Figure** **1**, there are several mainstream viewpoints for Fe–N–C active centers, including N–C groups, FeN*_x_* C*_y_* or Fe_2_N*_x_* C*_y_* moieties, iron oxide particles (FeO*_x_* (C*_y_*)) exposed in electrolyte, and iron particles embedded in N‐doped carbon (Fe@N–C).^[^ ^13^ ^]^ Comparatively speaking, the isolated monatomic and diatomic active centers possess superior reaction and degradation mechanism to other potential active sites. Specifically, Jaouen and collaborators reported that FeN*_x_* C*_y_* generally had stronger four‐electron path selectivity in oxygen‐saturated 0.1 m HClO_4_ electrolytes compared to Fe@N–C.^[^ ^14^ ^]^ The study by Maillard et al. showed that the isolated metal atoms in FeN*_x_* C*_y_* owned better stability in acidic media than that in Fe@N–C.^[^ ^15^ ^]^ Meanwhile, different significant issues exist for those infaust active sites, such as the severe carbon oxidation phenomenon in Fe@N–C, and low catalytic activity of N–C for ORR.^[^ ^14^ ^]^ Besides, the iron atom is easily detached from the FeO*_x_* (C*_y_*) site and fall into the electrolyte in the form of Fe^2+^, thus exhibiting poor stability.^[^ ^14^ ^]^ Therefore, ORR catalysts with FeN*_x_* C*_y_* or Fe_2_N*_x_* C*_y_* sites are more advantageous.^[^ ^13^, ^16^ ^]^

Theoretically, it can be up to at least five coordination modes for the FeN*_x_* site (such as FeN_2_, FeN_3_, FeN_4_, FeN_5_, FeN_6_) in Fe–N–C catalysts.^[^ ^17^ ^]^ With a template casting strategy, Guo's group designed and synthesized a N‐doped ordered mesoporous carbon materials with highly atomically dispersed FeN_2_ sites, which showed good ORR performance.^[^ ^17d^ ^]^ FeN_3_ sites usually are not ideal choice for ORR catalysts due to higher resistance of ORR.^[^ ^17c^ ^]^ In addition, the N atom as an axial ligand can also change the geometry and electronic structure of Fe centers in FeN_5_ configuration, and affect ORR performance.^[^ ^17e^ ^]^ Furthermore, the lower adsorption energy of FeN_5_ for *OH can effectively avoid damage of the catalytic sites.^[^ ^18^ ^]^ In addition, Su et al.^[^ ^17a^ ^]^ also constructed Fe^III^ coordination compounds with six coordination (FeN_6_). Although it contained more other phases, the catalyst still presented admirable catalytic activity and durability. However, due to the complexity of preparation toward pure samples, there are few studies on FeN_5_ and FeN_6_ at present.

Shui et al.^[^ ^19^ ^]^ compared a series of Fe–N–C catalysts containing FeN*_x_* sites with the same active site density and multiple coordination, and found that FeN_4_ had the superb ORR activity, the lowest formation energy and the outstanding PEMFC performance. In addition, a recent study by Li et al.^[^ ^20^ ^]^ demonstrated that FeN_4_ had a higher ability to prevent aggregation and demetallization of metal atoms than FeN_2_ and FeN_3_. The results of Bader charge analysis showed that the charges transferred from Fe atoms to N atoms were approximately equal. As a result, the structure of the catalyst (FeN_4_) with more coordination numbers of N atoms around Fe atoms anchored on the support was more stable.^[^ ^20^ ^]^ Therefore, most of the work on Fe–N–C catalysts are mainly around the Fe atom with four nitrogen coordination, namely FeN_4_.^[^ ^21^ ^]^ Jaouen's study showed that the Fe–N–C catalyst initially contained two different FeN_4_ sites: a high‐spin FeN_4_C_12_ part (S_1_) and a low‐ or medium‐spin FeN_4_C_10_ part (S_2_).^[^ ^22^ ^]^ Both sites initially contributed to the oxygen reduction reaction activity, but as the reaction time continued, S_1_ type was converted to iron oxide and degraded, while the structure and quantity of S_2_ type remained unchanged. Although S_1_ type does not have better stability, it tends to show higher catalytic activity than S_2_ type.^[^ ^23^ ^]^ Besides, the position of FeN_4_ active sites can significantly affect ORR performance.^[^ ^24^ ^]^ When FeN_4_ sites are located at the edge of the carbon plane, they tend to own better catalytic effects due to local electron redistribution and bandgap contraction.^[^ ^24b^ ^]^ Consequently, optimizing the electronic structure of FeN_4_ is a very significant method to improve the catalytic activity. This is because the electronic modulation can lead to redistribution of spin and charge density, enhancing catalytic performance.^[^ ^25^ ^]^ Generally, changing the close‐range collaborative environment and the long‐distance interaction with heteroatoms is an eventful means to improve the electronic structure of active centers.^[^ ^26^ ^]^

Finally, researchers have discovered that the diatomic sites (Fe_2_N*_x_*) can fruitfully compensate for the disadvantages of single‐atom sites.^[^ ^27^ ^]^ Although Fe–N–C catalysts with isolated FeN*_x_* sites have the highest atomic utilization rate and excellent catalytic activity, their catalytic activity center structure is so simple that they can only bond with single oxygen atom of intermediate product during oxygen reduction reaction.^[^ ^12^, ^16^, ^18^ ^]^ The resulting terminal adsorption state will lead to the high O—O bond breaking energy and reduce the catalytic activity of FeN*_x_* potential.^[^ ^28^ ^]^ Importantly, their active sites tend to isolate *OOH intermediates, resulting in side reactions that greatly lower the stability of the Fe–N–C catalyst. Thus, a catalyst with more active sites is more conducive to the improvement of stability.^[^ ^27a^ ^]^ The O_2_ adsorption configuration on the surface of the catalyst includes super oxygen or similar peroxy O_2_. After the peroxy‐type O_2_ is bonded to two adjacent Fe atoms, more electrons are provided to the empty O_2_ orbital, resulting in better activation.^[^ ^29^ ^]^ Holby's team evaluated the ORR performance of a series of Fe*_x_* N*_y_* sites via the density functional theory (DFT) calculation.^[^ ^30^ ^]^ The results showed that Fe_2_N_5_ can cleave the O_2_ bond with zero barrier, and possess very high selectivity, effectively avoiding the formation of H_2_O_2_ from the ORR intermediate. Interestingly, a catalyst with a planar Fe_2_N_6_ structure was synthesized by Xie et al., with an super oxygen adsorption capacity and a synergistic advantage of multiple accessible active sites.^[^ ^31^ ^]^ The catalyst possessed excellent driving forces for O—O bond fracture and ORR transformation, so revealing better catalytic activity with a highly efficient four‐electron transfer route.

As described above, Fe_2_N*_x_* seems to have more advantages over FeN*_x_* in terms of stability and activity. Even so, the preparation of such catalysts with uniform and highly dispersed diatomic sites often faces still challenges. In addition, diatoms tend to aggregate or form alloys, which often leads to degradation of catalytic performance.^[^ ^32^ ^]^ Besides, in some diatomic catalysts currently reported, there are often more single‐atom sites.^[^ ^33^ ^]^ This makes the identification of active sites or centers in the catalyst difficult. Therefore, developing the diatomic catalyst with more precisely control to the loading, bonding, and configuration of active sites is required.^[^ ^34^ ^]^

## 3 Probable Degradation Mechanisms

**Figure 2:**
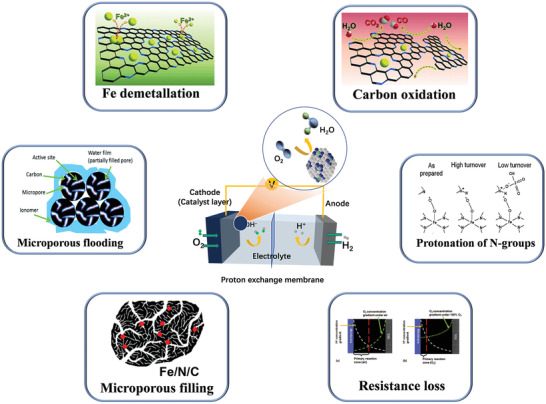
Possible decay mechanism of Fe–N–C catalyst performance. Images for “Fe demetallation and Carbon oxidation”: Reproduced with permission.^[^ ^51^ ^]^ Copyright 2015, Wiley‐VCH. Image for “Protonation of N‐groups”: Reproduced with permission.^[^ ^46^ ^]^ Copyright 2011, American Chemical Society. Image for “Resistance loss”: Reproduced with permission.^[^ ^56^ ^]^ Copyright 2017, Elsevier. Image for Micropores filling: Reproduced with permission.^[^ ^59^ ^]^ Copyright 2018, American Chemical Society. Image for “Micropores flooding”: Reproduced with permission.^[^ ^39b^ ^]^ Copyright 2017, Royal Society of Chemistry.

The decay of the catalytic performance of Fe–N–C catalysts can be divided into two sections: the rapid decrease of the catalytic activity at the beginning of the test, and then the slow and gradual decrease during the long‐term test.^[^ ^35^ ^]^ Thus, to keep the catalytic activity of the catalyst, it is important to avoid or slow down the decay process. However, due to the complexity of the ORR process and the uncertainty of the composition and microstructure of Fe–N–C catalysts, the degradation mechanism of the catalytic activity in the catalyst is still unclear. So far, as shown in **Figure** **2**, it is believed that the main reasons for the degradation related to active sites could be attributed to: 1) microporous flooding, 2) Fe demetallization, 3) protonation of N‐groups, 4) carbon corrosion, 5) resistance loss, and 6) micropore filling.

### 3.1 Microporous Flooding

The flooding of the catalyst layer often occurs at the cathode of PEMFCs, which greatly affects the performance of the catalyst.^[^ ^36^ ^]^ Dodelet and collaborators found that the slow oxidation of the carbon matrix in Fe–N–C catalysts used in H_2_/O_2_ fuel cells would cause the property of catalyst layers to change from hydrophobic to hydrophilic, resulting in the flooding of micropores, hindering the transport of O_2_ to the active site, and eventually resulting in rapid attenuation of catalyst performance.^[^ ^37^ ^]^ But in their recent studies, they clarified that the mechanism of the rapid degradation of catalyst performance was mainly not the hydrophilicity of the micropore walls aroused by electrooxidation, but the metal shedding from the FeN_4_ catalytic sites caused by the rapid water flow driven by the airflow through the catalyst layers.^[^ ^38^ ^]^ Whether it is the hydrophilicity of the micropore walls or the metal shedding, it can be identified by the recoverable or nonrecoverable activity of catalysts. In terms of water management, the activity can be recovered by mitigating the micropore blooding. However, it is nonrecoverable for metal loss at FeN_4_ catalytic sites. The actual effect of flooding of the catalyst layer on the degradation of catalyst performance is still controversial.^[^ ^39^ ^]^

### 3.2 Fe Demetallation

Early research on demetalization was mainly on Pt‐based catalysts.^[^ ^40^ ^]^ The structure and composition of Fe–N–C catalysts are more complex, so their decay mechanism is very different from that of Pt‐based catalysts. The Fe species at the catalyst can be corroded by hydroxyl radicals and leached out, thereby destroying the active site and degrading the membrane and ionomer.^[^ ^41^ ^]^ In detail, the leached iron will further promote the Fenton reaction and accelerate the degradation of the catalyst and membrane

(1) $$Fe^{2+}+H_{2}O_{2}\to Fe^{3+}+\mathrm{HO}\cdot +OH^{-}$$

(2) $$Fe^{3+}+H_{2}O_{2}\to Fe^{2+}+\mathrm{HOO}\cdot +H^{+}$$

In addition, free radicals can also attack the protective ligands at the active site, leading to more serious demetallization.^[^ ^42^ ^]^ Among the Fe species, atomic dispersion Fe sites (FeN*_x_* C*_y_*) are more resistant to demetallization than Fe@N–C.^[^ ^43^ ^]^ Duyne et al.^[^ ^44^ ^]^ observed the chemical changes of FePc during the ORR process in 0.1 m HClO_4_ by means of electrochemical tip‐enhanced Raman spectroscopy (EC‐TERS) and found that the main product after FePc degradation was free alkali phthalocyanine (H_2_Pc), confirming that the decrease in activity is related to the demetalization process. It is noted that the demetallization will lead to direct loss of Fe^2+^, and thus this process is irreversible.

### 3.3 Protonation of N‐Groups

The protonation of N‐groups in acid electrolytes also can contribute to reducing the catalyst activity.^[^ ^45^ ^]^ The N‐groups on the surface of the catalyst that cannot coordinate with Fe will be protonated quickly in acidic environment, causing the loss of catalytic activity.^[^ ^46^ ^]^ Generally, the degradation of the catalyst activity in polymer electrolytes is slower than that in liquid electrolytes, due to the different permeability of the anion in the electrolyte. In addition, heating or chemical means can remove the anion that binds to the N‐group, so that the part of lost activity of the FeN*_x_* site can be restored to some degree. However, a recent study revealed that strong basic N‐groups can accelerate demetallization of highly active FeN*_x_* sites, leading to irreversible performance loss.^[^ ^22^ ^]^ The pyrolysis of Fe–N–C precursors under NH_3_ tends to introduce strongly basic N‐groups, which could boost the turnover frequency (TOF) of the active sites, but it would reduce the durability of the catalyst.^[^ ^47^ ^]^ In an acidic electrolyte, compared to the Fe–N–C catalyst obtained by pyrolysis in Ar, the Fe–N–C catalyst obtained in NH_3_ atmosphere has a higher demetallization rate.^[^ ^48^ ^]^ In alkaline media, their demetallization rates are similar. A reasonable explanation is that the strong basic N‐group makes the protonation rapid in acidic media, which causes the rapid demetalization of FeN*_x_* sites with high TOF.^[^ ^48^ ^]^

### 3.4 Carbon Corrosion

The corrosion of the carbon matrix is considered to be a major cause of the large‐scale decay of catalytic performance. According to the carbon oxidation kinetics, the carbon corrosion in PEMFCs is due to the surface chemical reaction with O_2_, hydrogen peroxide or other reactive oxygen species (ROS) produced by the side reaction of ORR to produce CO or CO_2_^[^ ^49^ ^]^

(3) $$C+\;O_{2}\to CO_{2}$$

(4) $$2C+\;O_{2}\to 2\mathrm{CO}$$

Besides, the carbon corrosion often occurs during the startup/shutdown process cycle where the carbon support at the cathode can react with H_2_O under high potentials^[^ ^50^ ^]^

(5) $$C+2H_{2}O\to CO_{2}+4H^{+}+4e^{-}$$

(6) $$C+H_{2}O\to \mathrm{CO}+2H^{+}+2e^{-}$$

The degradation mechanism of Fe–N–C catalysts was analyzed with differential electrochemical mass spectrometry (DEMS) and online inductively coupled plasma mass spectrometry (ICP‐MS), the results presented that corrosion of the carbon matrix occurred at a high potential (> 0.9 V), and iron leaching occurs at a low potential (<0.7 V).^[^ ^51^ ^]^ The latest research clearly showed that even very low potential cycles in the presence of oxygen can cause carbon corrosion and thus loss of FeN*_x_* sites.^[^ ^52^ ^]^ By exposure to H_2_O_2_, the precise FeN*_x_* C*_y_* moieties of the structure remains unchanged, but the corrosion of the carbon surface reduces its switching TOF and then attenuates the O_2_ binding force at the site.^[^ ^53^ ^]^ Choi et al.^[^ ^54^ ^]^ further demonstrated that the carbon corrosion can aggravate the occurrence of demetallization under acidic conditions, allowing the Fenton reaction to further accelerate the corrosion of the carbon matrix.

### 3.5 Resistance Loss

**Figure 3:**
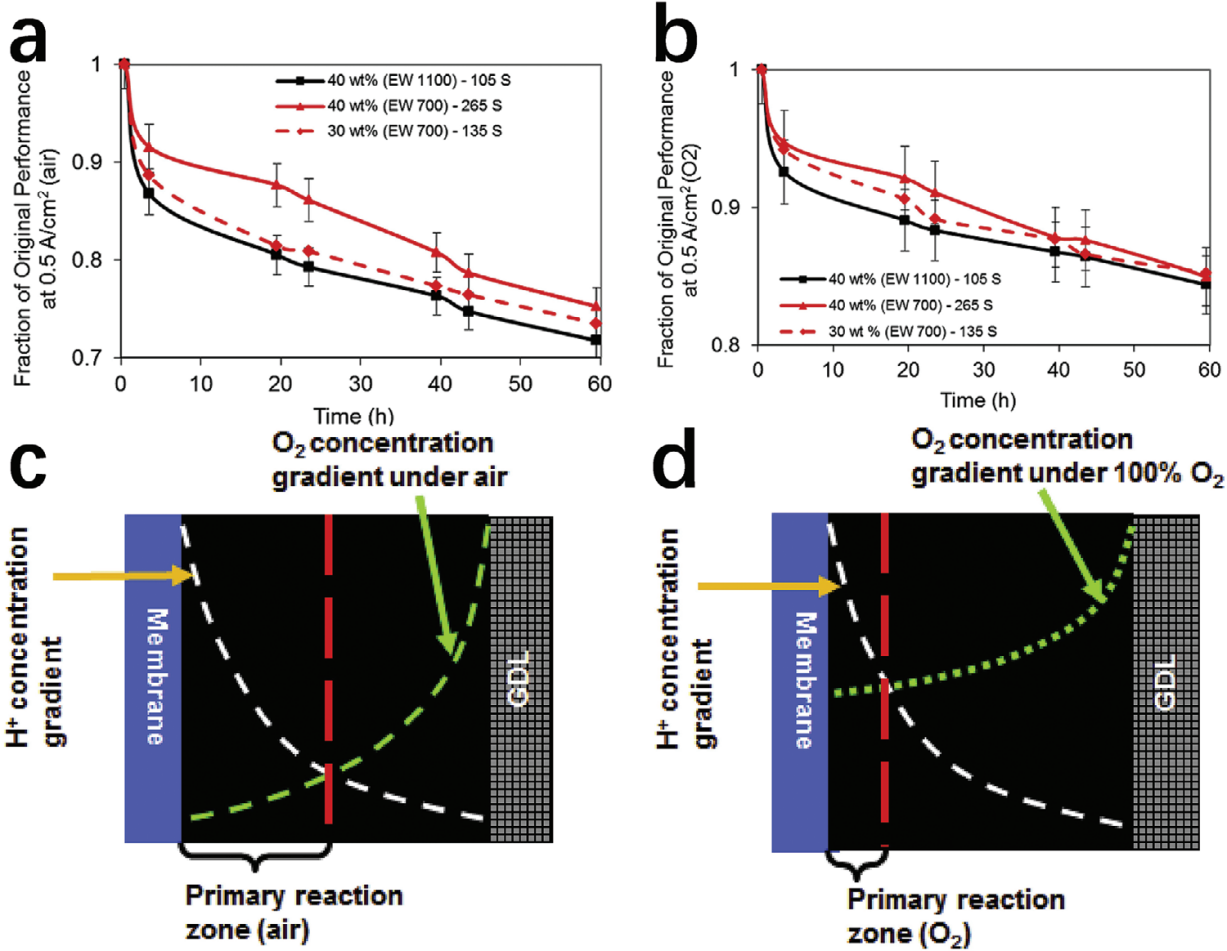
a) Stability of the Fe–N–C catalyst layer under H_2_/air. b) Long‐term tests of the Fe–N–C catalysts layer under H_2_/O_2_. c) Schematic diagram of the proton transport distances under air. d) Schematic depicting the proton transport distances under O_2_. a–d) Reproduced with permission.^[^ ^56^ ^]^ Copyright 2017, Elsevier.

Resistance loss is a related factor in the degradation of the catalyst layer. Nonprecious‐metal ORR catalyst layers are often thick, which can cause serious mass transfer problems.^[^ ^47^, ^55^ ^]^ Although it reduces porosity, ionomers play a central role in the thick catalyst layer structure due to their excellent proton conductivity. Banham et al.^[^ ^56^ ^]^ used 1100 and 700 equivalent weight (EW) ionomer designs to evaluate the stability of the catalyst layer in H_2_/O_2_ and H_2_/air fuel cells, respectively. They found that the stability of high EW ionomers in the air was worse than that of low EW ionomers, while their stability is similar in a pure oxygen environment (**Figure** **3**a,b). One hypothesis is that the loss of the active site mainly occurs at the interface between the catalyst layer and the membrane, causing the position where the oxygen reduction reaction occurs to gradually move further away from the membrane (Figure 3c,d). The attenuation of the active site inevitably results in a loss of kinetics. The protons must go deep into the catalyst layer, causing more forms of loss. In addition, complete deactivation of the catalyst at the interface would cause ohmic losses, and partial deactivation could trigger nonohmic losses.

### 3.6 Micropores Filling

**Figure 4:**
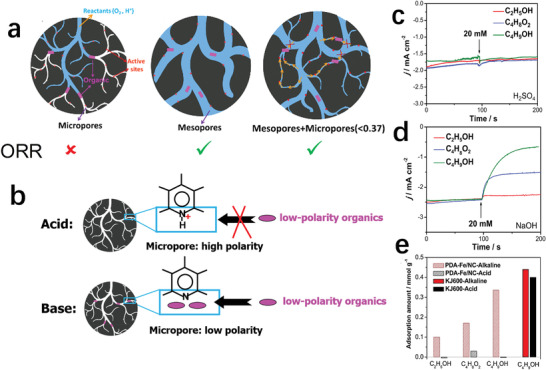
a) Schematic diagram of catalysts with different structures mixed into small organic molecules. b) Schematic depicting the structure of catalysts mixed with small organic molecules in different electrolytes. c) Chronoamperometric responses of Fe–N–C catalysts before and after dropping 20 × 10^−3^ m organic solvent in 0.1 m H_2_SO_4_ at 0.80 V. d) *I*–*t* curves of Fe–N–C catalysts before and after dropping 20 × 10^−3^ m organic solvent in 0.1 m NaOH at 0.80 V. e) The adsorption capacity of different organic molecules on PDA–Fe–N–C and KJ600 carbon black in 0.1 m NaOH and 0.1 m H_2_SO_4_ solutions. a–e) Reproduced with permission.^[^ ^59^ ^]^ Copyright 2018, American Chemical Society.

The oxygen used in fuel cells is often unavoidably mixed with some small organic molecules (SOMs), therefore, excellent resistance to poisoning by SOMs is an important aspect of high‐performance ORR catalysts.^[^ ^57^ ^]^ Many SOMs tend to be easily adsorbed on the surface of Pt and oxidized, which reduces the ORR catalytic activity of the active site.^[^ ^58^ ^]^ Therefore, the current commercial Pt/C catalysts often have poor resistance to SOMs. It is widely believed that Fe/N/C catalysts have excellent resistance to poisoning by SOMs. However, as shown in **Figure** **4**a, a study by Sun's group showed that methanol and ethanol had a significant inhibitory effect on the ORR process of Fe–N–C catalysts rich in micropores (PDA–Fe–N–C) in alkaline environment.^[^ ^59^ ^]^ Through the investigation of the size, polarity and inhibition ability of organic molecules, it was found that the inhibitory effect of low polarity and larger SOMs on the catalyst performance was more obvious (Figure 4c–e). Therefore, the reason for the inhibition is believed to be that the filling of the micropores by the SOMs leads to the decrease of the mass transfer capacity. In addition, this suppression phenomenon did not occur in acidic environment. This may be due to the protonation of the N‐group, which changes the polarity of the micropores and reduces the adsorption of SOMs (Figure 4b).

## 4 Improvement Strategies for Stability

Generally, achieving highly stable Fe–N–C catalysts with wonderful activity is greatly expected yet challenging. In Section 2, we summarize and compare the different types of active sites (centers) of Fe–N–C catalysts in terms of stability. In addition to the precise design of Fe–N–C active sites, it is very necessary to further stabilize them through external strategies to avoid or slow down the performance degradation of such catalysts. Some strategies and methods have been raised to enhance the stability of the catalyst, including strengthening the electronic coupling, avoiding the attack of H_2_O_2_, increasing the oxidation resistance of the carbon substrate and rational design of the catalyst layer.

### 4.1 Strengthening the Electronic Coupling

Enhancing the electronic coupling between the carbon matrix and the active sites is an effective means to prevent Fe demetallization. Previous studies revealed that the FeN*_x_* C*_y_* moieties of the catalyst was not prone to Fe removal and exhibits excellent stability.^[^ ^43^ ^]^ Many researchers have devoted themselves to the development of electrocatalysts only containing FeN*_x_* C*_y_*.^[^ ^60^ ^]^ Otherwise, due to the huge influence of Fe demetallization on the performance of PEMFC, it is necessary to further improve the electronic coupling between the carbon matrix and the active sites, mainly including introducing substituents, and adjusting the active site distribution and annealing temperature.

**Figure 5:**
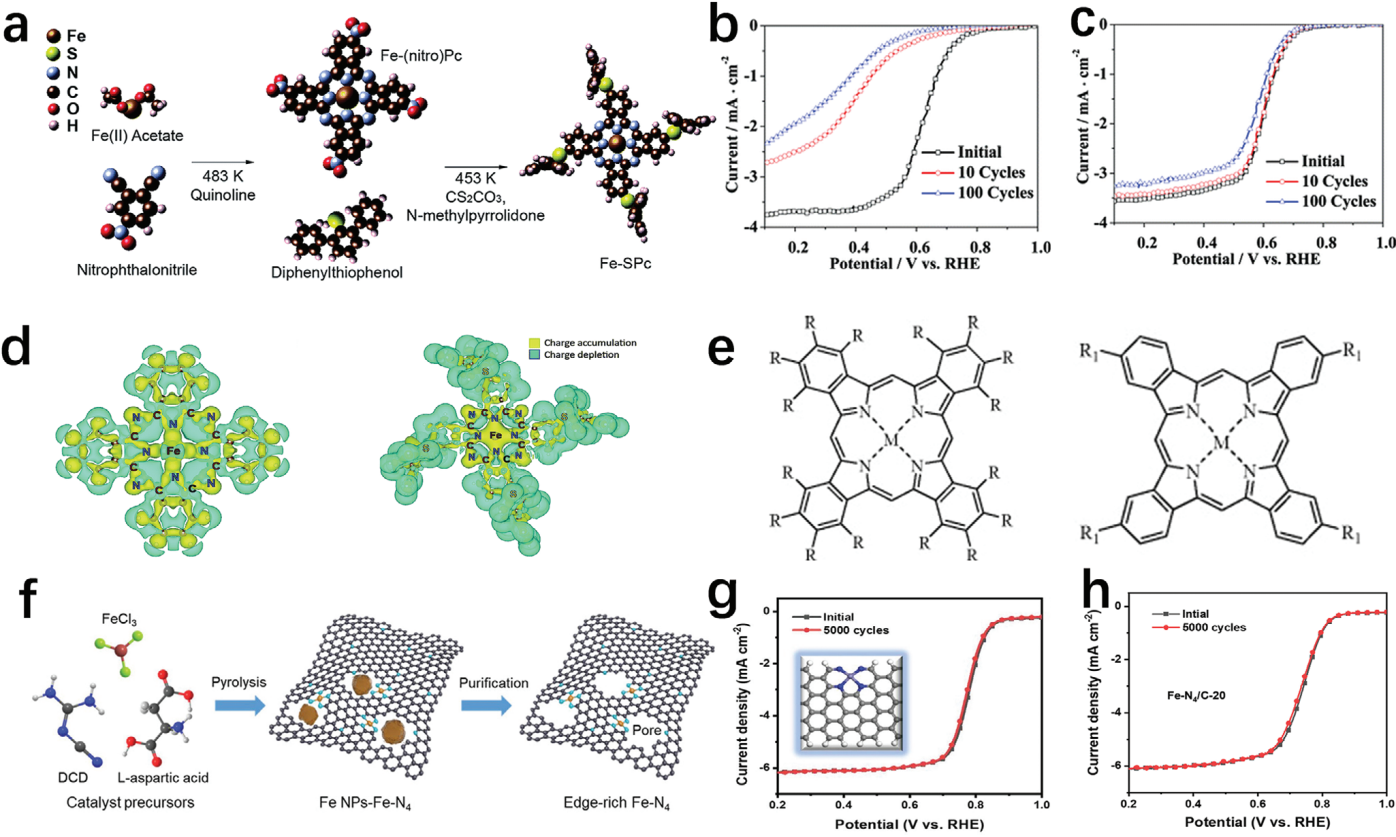
a) Schematic diagram of Fe–SPc synthesized from Fe–Pc. Reproduced with permission.^[^ ^62^ ^]^ Copyright 2014, Royal Society of Chemistry. b) The accelerated durability test of Fe–Pc. c) The LSV curves of Fe–SPc before and after 10 and 100 cycles. b,c) Reproduced with permission.^[^ ^61^ ^]^ Copyright 2010, American Chemical Society. d) Charge density changes of Fe–Pc and Fe–SPc. Reproduced with permission.^[^ ^62^ ^]^ Copyright 2014, Royal Society of Chemistry. e) A molecular structure of modified Fe–Pc: R = Cl or F; R_1_ = tBu, Me, NO_2_, NH_2_, or F, Cl. Reproduced with permission.^[^ ^63^ ^]^ Copyright 2016, Elsevier. f) Synthesis of Fe–N–C catalyst followed by its carbonization to get edge‐rich carbon. g) Accelerated durability test of Fe–N_4_/C‐60. h) ORR polarization plots of Fe–N_4_/C‐20 before and after multiple cycles. f–h) Reproduced with permission.^[^ ^24b^ ^]^ Copyright 2020, Wiley‐VCH.

The introduction of substituents to tune the electronic structure of the catalytic site can validly slow down the metals removal.^[^ ^42^ ^]^ Chen et al.^[^ ^61^ ^]^ modified ferrous phthalocyanine (Fe–Pc) with diphenylphenyl sulfide substituents to prepare a highly stable oxygen reduction catalyst (Fe–SPc) (**Figure** **5**a). The performance of Fe–PC and Fe–SPC in oxygen‐saturated 0.1 m HClO_4_ was evaluated using a rotating disk electrode (RDE). Compared to Fe–Pc, although the catalytic activity of Fe–SPc slightly decreased, its stability was greatly improved (Figure 5b,c). After 10 and 100 scanning cycles, the current density of Fe–SPc at 0.5 V versus RHE remained 4.6 and 7.4 times that of Fe–Pc, respectively. DFT calculations about the electronic structure of Fe–SPc and Fe–Pc disclosed the relative upward movement of the $d_{z}^{2}$ orbital position of the iron atom in Fe–SPc.^[^ ^62^ ^]^ This resulted in a stronger oxygen intermediate bond, and then a slight decrease in ORR activity. But compared to Fe–Pc, the cohesive energy of FeO to Fe–SPc increased, conducive to improving stability. Moreover, the changes in the charge density of the two sites indicated that their charges were symmetrically distributed, and the charges were concentrated around nitrogen (N) atoms (Figure 5d). In addition, the central Fe ion in Fe–Pc showed charge depletion, and the dithiophenol group on Fe–SPc could donate electrons to form accumulated charges. The electron‐donating group of diphenylthiophenol substituted on the phthalocyanine complex could effectively slow down the demetalization of the active sites by donating additional electrons to the Fe center. Therefore, Fe–SPc had better electrochemical stability than Fe–Pc. Chen and co‐workers further explored the influence of electron‐withdrawing and electron‐donating substituents on the stability of Fe–Pc (Figure 5e).^[^ ^63^ ^]^ The results showed that FePc with electron‐donating substituents (Me‐, NH_2_‐, tBu‐) had a lower demetallization percentage than the parent compound, indicating that the stability of FePc can be enhanced by introducing electron‐donating substituents into the macrocycle. The electron withdrawing substituents such as 4Cl‐, 4F‐, and 16Cl‐ could enhance the stability of FePc to a certain extent. However, strong electron‐withdrawing substituents including 16F‐ and 4NO_2_‐ would reduce the stability of FePc.

Additionally, by comparing the defect formation energy between the edge and the body of carbon matrix, it could be found that the defect formation energy of the FeN_4_ site at the edge structures was higher than that on the inside.^[^ ^64^ ^]^ This indicated that the Fe–N defects located at the edge of the nanoribbons tend to be more stable than the Fe–N defects located at the inner part of the nanoribbons. It is reported that Fe–N defects near the pore structure had a similar stabilizing effect, which is probably due to the relax carbon structure near the open edge.^[^ ^64^ ^]^ DFT calculation demonstrated that, compared to the complete N‐modified divacancies in the center of the carbon plane trapped Fe sites (c‐ND–Fe), the local electron redistribution and bandgap contraction of N‐modified divacancies at the edge of the carbon plane trapped Fe sites structure (e‐ND–Fe) would reduce four‐electron ORR free energy. Therefore, fixing FeN*_x_* at the edge of carbon plane to construct catalytic sites is helpful to improve the stability and activity of the catalyst. Yao et al.^[^ ^24b^ ^]^ synthesized a series of Fe–N–C catalysts with different proportions of e‐ND–Fe (Fe–N_4_–C‐*x*) by adjusting the content of FeCl_3_ in the precursor (Figure 5f), and proved that the catalytic performance was extremely dependent on the part of e‐ND–Fe. The FeN_4_ sites of Fe–N_4_/C‐20 were mainly located inside the carbon plane, while they were on the edge for Fe–N_4_/C‐60. The accelerated cyclic voltammetry test in O_2_‐saturated 0.1 m HClO_4_ aqueous solutions using RDE showed that the LSV curve of Fe–N_4_/C‐20 changed little after 5000 cycles (Figure 5h). But more prominently, the LSV curves of Fe–N_4_/C‐60 before and after the accelerated test almost overlapped, showing a better durability (Figure 5g). Moreover, the pyrolysis temperature has an obvious influence on the bond strength and bond length of Fe—N bonds. Wu and co‐workers explored the relationship between the bond length/ bond strength of the intrinsic Fe—N bond and the temperature. The result confirmed that the bond strength of the Fe—N bond was the highest at 700 °C.^[^ ^65^ ^]^ With further increased temperatures, it would lead to nitrogen shedding, causing the reduction of activity.

### 4.2 Avoiding the Attack of H_2_O_2_

In the ORR process, the attack from H_2_O_2_ and ROS produced by the side reaction is the main reason for the performance degradation of TM–H–C catalysts.^[^ ^66^ ^]^ Therefore, to protect the carbon substrate and active sites, and prevent the deterioration of the catalyst, avoiding the attack of H_2_O_2_ is of great significance for the catalyst. The main methods are to enhance four‐electron path selectivity from the source and quickly eliminate generated H_2_O_2_ and ROS.

#### 4.2.1 Enhancing Four‐Electron Path Selectivity

**Figure 6:**
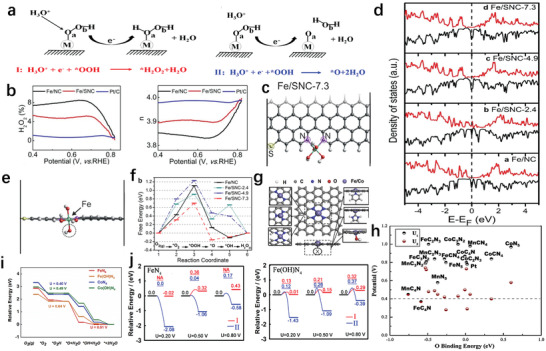
a) Schematic depicting the reaction from *OOH to *H_2_O_2_ or H_2_O. Reproduced with permission.^[^ ^73^ ^]^ Copyright 2020, Elsevier. b) Hydrogen peroxide production and electron transfer number of different catalysts. c) Optimized geometry of Fe/SNC‐7.3 in the horizontal direction. d) Density of states of different catalysts. e) Optimized geometry of Fe/SNC‐7.3 in the vertical direction. f) The thermodynamic activity of the Fe/NC with and without S during the ORR at an equilibrium potential of U = 1.23 V. b–f) Reproduced with permission.^[^ ^70^ ^]^ Copyright 2017, Wiley‐VCH. g) Diagrammatic sketch of in‐plane and axial regulation of metal centers of FeN_4_. Reproduced with permission.^[^ ^73^ ^]^ Copyright 2020, Elsevier. h) U_1_ and U_2_ of different ORR catalysts. Reproduced with permission.^[^ ^67^ ^]^ Copyright 2016, Elsevier. i) Free‐energy diagrams for FeN_4_, Fe(OH)N_4_, CoN_4_, and Co(OH)N_4_. j) Reaction energy and kinetic barrier of *OOH reduction under different voltages in FeN_4_ and Fe(OH)N_4_ models. i,j) Reproduced with permission.^[^ ^73^ ^]^ Copyright 2020, Elsevier.

As the most earthly ORR catalysts to substitute commercial Pt/C, the improvement of the four‐electron pathway selectivity of Fe–N–C catalysts is meaningful. For the ORR catalyst, the sticking point of reducing the production of H_2_O_2_ is to accelerate the four‐electron reduction and suppress the two‐electron reduction.^[^ ^67^ ^]^ The ORR catalyst with too weak oxygen adsorption capacity is usually difficult to form hydroxyl intermediates on the surface. Excessive oxygen adsorption capacity would make the H_2_O_2_ combined with *OOH and *H difficult to leave, while not conducive to the removal of H_2_O formed by the combination of *OH and *H. Generally, the Fe–N–C catalyst has a strong oxygen adsorption capacity, which is not conducive to its four‐electron selectivity.^[^ ^68^ ^]^ In addition, promoting the break of O—O bond in *OOH to inhibit the generation of hydrogen peroxide is also an important aspect of improving the four‐electron selectivity (**Figure** **6**a).^[^ ^69^ ^]^ Therefore, the key to improving the four‐electron pathway selectivity of the Fe–N–C catalyst is to adjust the oxygen adsorption capacity to an appropriate level and promote the break of O—O bond in *OOH. The strategies to boost the four‐electron pathway selectivity of Fe–N–C catalysts mainly include: heteroatoms doping, adjustment of axial coordination and construction of dinuclear sites.

Through the doping of heteroatoms with different electronegativity, the spin density and local charge of Fe in Fe–N–C catalyst will be changed, thereby adjusting the ability to absorb oxygen and improving the four‐electron path selectivity of the catalyst.^[^ ^55c^ ^]^ Guo's team demonstrated that the Fe–N–C catalyst doped with a certain amount of sulfur in the carbon framework (Fe/SNC‐7.3) had a higher electron transfer rate and a lower H_2_O_2_ yield than the undoped sample in O_2_‐saturated 0.5 m H_2_SO_4_ (Figure 6b).^[^ ^70^ ^]^ They used DFT calculations to analyze this phenomenon and proposed that the unique position of the sulfur atom allowed greater availability of electronic states near the Fermi level, thereby lowering the electron positioning near the iron center and reducing oxygen adsorption capacity (Figure 6c–e). As shown in Figure 6f, the strong interaction of the catalyst with *O_2_ and *OOH intermediates enabled a complete 4 e^−^ ORR to be achieved with a reduced activation energy barrier. Moreover, the strong adsorption energy of Fe/SNC‐7.3 on H_2_O_2_ indicated its ability to complete H_2_O_2_ reduction if necessary.

Fe atoms have a large number of empty *d*‐bands perpendicular to the FeN*_x_* plane, so can coordinate with many groups to adjust the electronic structure of the FeN*_x_* catalyst (Figure 6g).^[^ ^71^ ^]^ Cho et al.^[^ ^67^ ^]^ developed two parameters U_1_ and U_2_ to characterize reaction trend. The U_1_ is the highest potential at which all steps in the four‐electron reduction reaction are downhill. The U_2_ is the lowest (highest) potential at which all reaction steps in the two‐electron reduction path are uphill (downhill). Compared to other active sites, FeN_5_ formed by pyrrole‐N as the axial coordination of FeN_4_ has a higher U_1_ value and a lower U_2_ value, indicating an excellent ability to accelerate four‐electron reduction and inhibit two‐electron reduction (Figure 6h).^[^ ^67^ ^]^ In addition, some studies have also shown that the use of OH ligands can obviously enhance the catalytic performance of the catalyst (Figure 6i).^[^ ^72^ ^]^ However, the investigation by Hu team showed that after Fe atom coordinated with the OH ligand, the forming Fe(OH)N_4_ would make the break of the O—O bond of *OOH more difficult and increase the production of H_2_O_2_.^[^ ^73^ ^]^ The O—O bond break on Fe(OH)N_4_ to form *O and H_2_O had almost equal barriers to the formation of H_2_O_2_ (Figure 6j). This means that the catalyst would produce a large amount of H_2_O_2_ during ORR, which seriously threatens the stability of the Fe–N–C catalyst. Some studies suggested that FeN_4_ sites could be converted to Fe(OH)N_4_ during ORR, making the problem of hydrogen peroxide generation more serious.^[^ ^72a^ ^]^ Although common RRDE tests exhibit that the H_2_O_2_ yield of the catalyst is very small, this is due to the limitations of the test method. Most of the hydrogen peroxide produced at the active site undergoes further reduction in the carbon framework and cannot be detected by the electrode.

**Figure 7:**
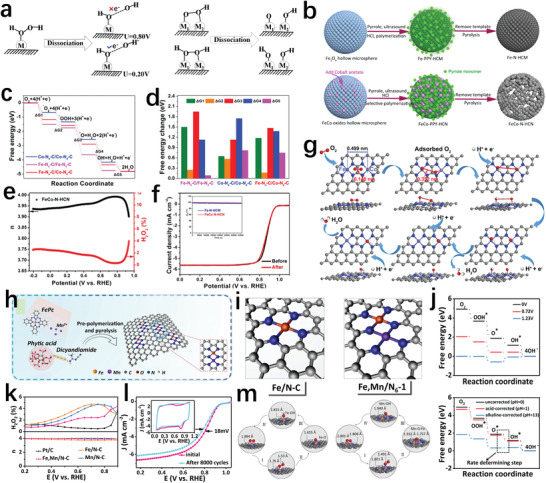
a) The adsorption mode of O_2_ molecules on single nuclear sites and binuclear sites. Reproduced with permission.^[^ ^73^ ^]^ Copyright 2020, Elsevier. b) Schematic diagram of preparation of Fe–N–HCM and FeCo–N–HCN. c) The pathways for different active sites. d) Energy changes for the ORR pathway. e) Number of electrons transferred and H_2_O_2_ production of FeCo–N–HCN. f) Stability tests of FeCo–N–HCN. g) Mechanism for ORR on the Fe–N_4_ and Co–N_4_ dual active centers. b–g) Reproduced with permission.^[^ ^76^ ^]^ Copyright 2021, Wiley‐VCH. h) Schematic illustration of the synthesis and structure of Fe, Mn/N–C. i) The optimized structure of Fe/N–C and Fe, Mn/N–C. j) The pathways for Fe, Mn/N–C. k) Electron transfer number and H_2_O_2_ yield of different catalysts. l) LSV curves and CV curves of Fe, Mn/N–C before and after 8000 cycles. m) The main process of ORR at different sites. h–m) Reproduced under the terms of the CC‐BY Creative Commons Attribution 4.0 International license (https://creativecommons/licenses/by/4.0).^[^ ^68^ ^]^ Copyright 2021, The Authors, published by Springer Nature.

In Section 2, the advantages of Fe diatomic sites in improving the selectivity of the four‐electron pathway of the catalyst are described. Moreover, as shown in **Figure** **7**a, binuclear sites composed of different metal atoms also can adjust the oxygen adsorption mode of the active center from an end‐on or side‐on model to a bridge‐*cis* model which is more conducive to O—O bond cleavage.^[^ ^29^, ^74^ ^]^ In addition, the binuclear sites composed of Fe atoms and other metal atoms are also helpful to weaken the excessive adsorption of Fe atoms to oxygen‐containing intermediates.^[^ ^68^ ^]^ Therefore, the introduction of other metal atoms to construct dinuclear sites can more effectively improve the four‐electrons path selectivity of the catalyst.^[^ ^68^, ^75^ ^]^ Luo et al.^[^ ^76^ ^]^ constructed an ORR catalyst with adjacent FeN_4_ and CoN_4_ as dual active centers (FeCo–N–HCN) (Figure 7b). When O_2_ molecules were adsorbed on such binuclear site, the O—O bond length greatly increased, allowing it easier to break and inhibit the formation of hydrogen peroxide (Figure 7c,d). Moreover, the *OH intermediate was finally adsorbed on the CoN_4_ site, preventing the FeN_4_ site from adsorbing oxygen molecules too strongly, and resulting in a smaller overpotential (Figure 7g). Therefore, FeCo–N–HCN exhibited highly efficient four‐electron transfer route and extremely low H_2_O_2_ production (Figure 7e). The stability curves in O_2_‐saturated 0.1 m KOH also confirmed the stability advantage of this Fe,Co binuclear site (Figure 7f). To effectually solve the problems of insufficient adsorption capacity of Mn–N–C for O_2_ and excessive adsorption capacity of Fe–N–C, Yang et al.^[^ ^68^ ^]^ constructed a binuclear Fe, Mn/N–C catalyst (Figure 7h,i). Such a catalyst could productively capture oxygen‐containing intermediates and break the M—OH bond, exhibiting excellent four‐electron pathway selectivity (Figure 7j,k,m). The accelerated cycle test in 0.1 m HClO_4_ solutions saturated with O_2_ showed the good stability for Fe,Mn/N–C (Figure 7l).

#### 4.2.2 Eliminating H_2_O_2_ and ROS

As aforementioned, H_2_O_2_ and ROS can severely corrode the carbon matrix and destroy the active sites, leading to decay of the Fe–N–C catalyst. The rapid elimination of these adversely affected intermediate products in the catalyst can effectively diminish the damage of such substances to the catalyst structure. The methods of rapid elimination mainly include the introduction of Pt and Ce‐based compounds.

**Figure 8:**
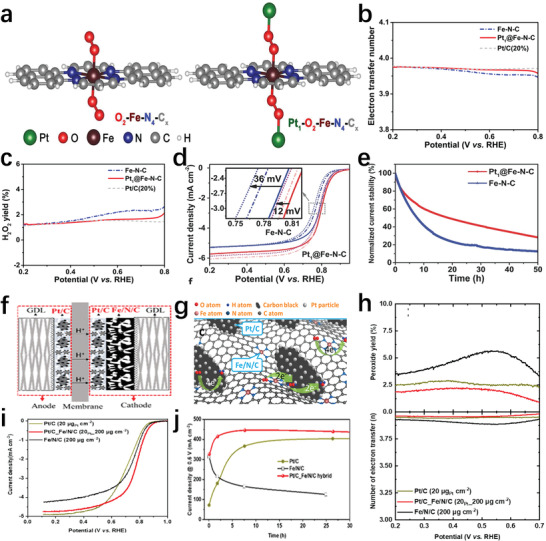
a) Molecular structure of O_2_–Fe–N_4_–C*_x_* and Pt_1_–O_2_–Fe_1_–N_4_–C*_x_*. b) Electron transfer number of Fe–N–C and Pt_1_@Fe–N–C. c) H_2_O_2_ yield before and after Fe–N–C catalyst grafting. d) The accelerated durability test. e) Stability test of MEAs with Fe–N–C and Pt_1_@Fe–N–C at 0.5 V with 80 °C H_2_/O_2_. a–e) Reproduced with permission.^[^ ^77^ ^]^ Copyright 2018, Wiley‐VCH. f) Schematic diagram of the MEAs with Pt/C and Fe–N–C hybrid cathodes. g) Schematic diagram of the surface reaction of the layered hybrid catalyst. h) Percentage of peroxide and electron transfer number of all catalysts. i) LSV curves of different catalysts. j) Stability curves of the MEAs made by the optimized hybrid cathode. f–j) Reproduced with permission.^[^ ^78^ ^]^ Copyright 2020, American Chemical Society.

The introduction of Pt into the Fe–N–C system is a very effective means to quickly quench hydrogen peroxide and active oxygen, which prevents the formed intermediate products from destroying the catalyst structure. Zeng et al.^[^ ^77^ ^]^ developed a kind of Fe–N–C catalysts with reconstructed Pt_1_–O_2_–Fe–N_4_–C*_X_* (Pt_1_@Fe–N–C) by grafting the Pt atom to the FeN_4_ accurately via bridging O_2_ molecules (**Figure** **8**a). Compared to Fe–N–C, the prepared Pt_1_@Fe–N–C had a smaller change in the LSV curve measured after 5000 and 10 000 voltage cycles in air‐saturated 0.5 m H_2_SO_4_ (Figure 8d). The durability of membrane electrode assemblies (MEAs) with such catalysts was obviously improved by the H_2_/O_2_ fuel cell test (Figure 8e). The Pt_1_–O_2_–cap could effectively improve the four‐electron path selectivity of the catalyst, which prevented Fe atoms from participating in the Fenton reaction and greatly reduced the concentration of hydroxyl radicals, avoiding further damage to the active sites and carbon support (Figure 8b,c). Sun et al.^[^ ^78^ ^]^ also simply prepared a hybrid Pt/C–Fe–N–C catalyst layer, which by simply covering a sufficiently thin Pt/C layer on the coated Fe–N–C layer (Figure 8f). The post‐coated Pt/C ink might penetrate the upper layer of the catalyst and the hot pressing process ensured complete contact between the two catalysts. The Fe–N–C catalyst with high porosity structure fully facilitated the dispersion of Pt, which avoided the generation of H_2_O_2_ and free radicals by the iron‐based active site nearby Pt (Figure 8g). The low hydrogen peroxide yield and the electron transfer number close to 4 indicated the high selectivity of the hybrid catalyst for the four‐electron path (Figure 8h). The LSV curves of Pt/C, Fe–N–C and Pt/C–Fe–N–C in 0.1 m HClO_4_ showed that Pt/C–Fe–N–C possessed the most excellent catalytic activity (Figure 8i). Consequently, compared to the pure Fe–N–C and Pt/C catalysts, the MEAs with such hybrid catalysts had shorter activation time and more stable current output (Figure 8j).

However, owing to the huge spending and the rarement of Pt, the application of above strategy is quite difficult. As an alternative to Pt, Ce^3+^ / Ce^4+^ redox can be used to effectively absorb H_2_O_2_ and scavenge free radicals

(7) $$Ce^{4+}+H_{2}O_{2}\to Ce^{3+}$$

(8) $$Ce^{4+}+\mathrm{HOO}\cdot \to Ce^{3+}$$

(9) $$Ce^{3+}+O_{2}+\mathrm{HO}\cdot +H^{+}\to Ce^{4+}+H_{2}O$$

**Figure 9:**
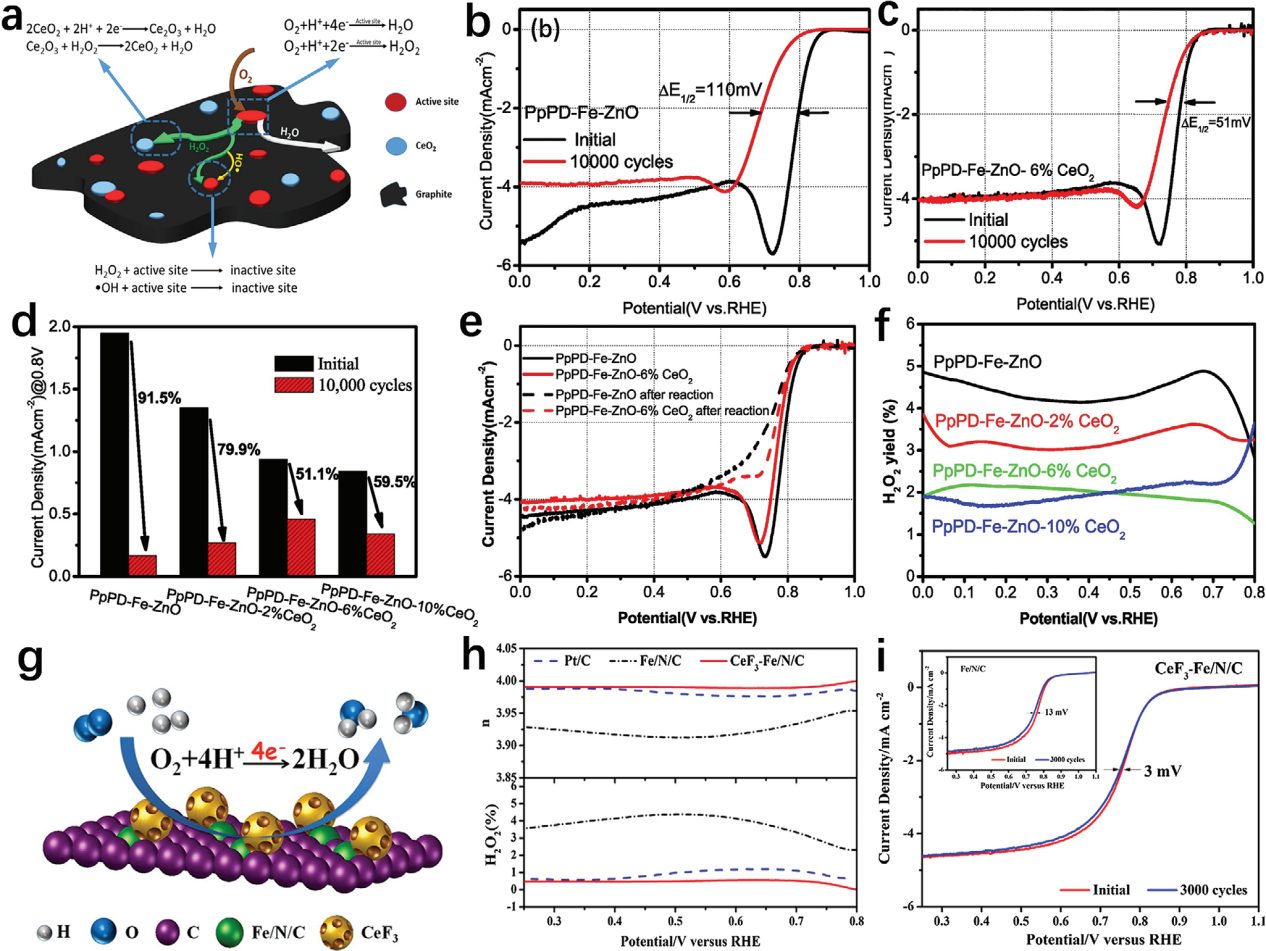
a) Proposed structural model of surface of PpPD–Fe–ZnO–6%CeO_2_. b) Linear sweep voltammetric curves before and after 10 000 cycles of PpPD–Fe–ZnO. c) The accelerated durability test of PpPD–Fe–ZnO‐6%CeO_2_. d) Current density of the catalysts at 0.8 V before and after 10 000 cycles. e) LSV curves of the catalysts before and after Fenton's reaction. f) The yield of H_2_O_2_ of all catalysts in ORR. a–f) Reproduced with permission.^[^ ^80^ ^]^ Copyright 2018, Elsevier. g) Oxygen reduction reaction on the surface of CeF_3_–Fe–N–C. h) Number of charge transfer and percentage of peroxide of all catalysts. i) LSV curves of CeF_3_–Fe–N–C and Fe–N–C before and after accelerated durability tests. g–i) Reproduced with permission.^[^ ^81^ ^]^ Copyright 2019, Elsevier.

In past studies, CeO_2_ was widely reported for quenching or removing H_2_O_2_ and ROS in Pt‐based catalysts.^[^ ^79^ ^]^ Therefore, it is worthwhile to introduce CeO_2_ into the Fe–N–C system to prevent the damage of the catalyst structure from hydrogen peroxide. To eliminate H_2_O_2_ and improve the stability of the Fe–N–C catalyst, Zou's group added CeO_2_ into Fe–N–C catalysts using zinc oxide as template (PpPD–Fe–ZnO–6%CeO_2_) (**Figure** **9**a).^[^ ^80^ ^]^ The accelerated durability test in a O_2_‐saturated 0.1 m HClO_4_ solution using RDE indicated that the stability of the catalyst was significantly improved after the introduction of CeO_2_ (Figure 9b–d). The hydrogen peroxide production graph and LSV curves before and after dropping the Fenton reagent containing H_2_O_2_ and FeSO_4_·7H_2_O further confirmed the ability of the Fe–N–C catalyst containing CeO_2_ to efficiently reduce H_2_O_2_ (Figure 9e,f). This is because CeO_2_ can effectively reduce the attack from hydrogen peroxide on the active sites and the oxidation of the carbon matrix, thereby giving the catalyst better stability. In addition to CeO_2_, CeF_3_ also has a similar effect on improving Fe–N–C catalyst stability. Yang and collaborators prepared a CeF_3_/Fe single atom hybrid catalyst (CeF_3_–Fe–N–C) (Figure 9g).^[^ ^81^ ^]^ The strong chemical interaction between CeF_3_ and Fe–N–C effectively regulated the surface state of Ce and Fe species. The catalyst structure introduced with CeF_3_ makes the oxygen‐philic interface more conducive to the adsorption of oxygen. More importantly, such catalyst performed ex situ chemical treatment to effectively adsorb and convert H_2_O_2_, which significantly boosts the ORR stability. The electrochemical test results in 0.5 m H_2_SO_4_ aqueous solutions saturated with O_2_ exhibited that the introduction of CeF_3_ could significantly increase the number of transferred electrons of the Fe–N–C catalyst and reduce the production of hydrogen peroxide (Figure 9h). Therefore, the stability of CeF_3_–Fe–N–C was also higher than pure Fe–N–C (Figure 9i).

### 4.3 Increasing the Oxidation Resistance of Carbon Substrate

As one of the most vital causes for performance degradation of catalysts, carbon corrosion occurs widely in carbon‐based catalysts due to oxidation. In view of the adverse effects of carbon corrosion on the stability of Fe–N–C catalysts, it is necessary to avoid the carbon substrate oxidation. Methods to increase the oxidation resistance of carbon substrate mainly embody the carbon graphitization and the edge regulation.

#### 4.3.1 Carbon Graphitization

**Figure 10:**
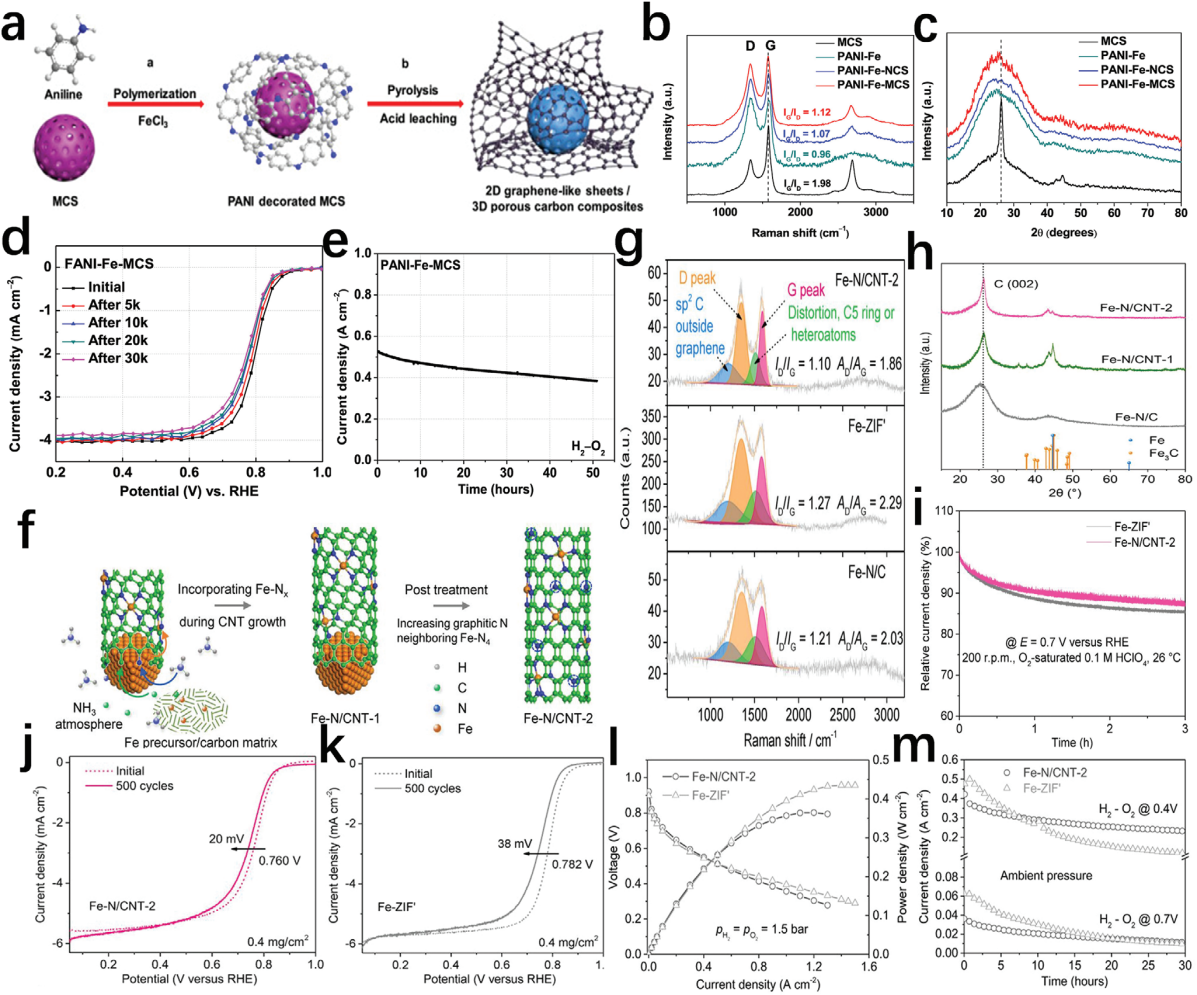
a) Schematic illustration of the synthesis and structure of PANI–Fe–MCS. b) Raman spectra of MCS, PANI–Fe, PANI–Fe–NCS, and PANI–Fe–MCS. c) XRD spectra of all catalysts. d) The accelerated durability test of PANI–Fe–MCS. e) Stability of PANI–Fe–MCS catalyst assembled as a fuel cell at 0.4 V. a–e) Reproduced with permission.^[^ ^85^ ^]^ Copyright 2017, Elsevier. f) Schematic illustration of a formation process for Fe–N/CNT‐2. g) XRD spectra of Fe–N/C, Fe–N/CNT‐1, and Fe–N/CNT‐2. h) Raman spectra of Fe–N/C, Fe–ZIF’, and Fe–N/CNT‐2. i) *I*–*t* curves of Fe–ZIF’ and Fe–N/CNT‐2. j) Accelerated stress test of Fe–N/CNT‐2. k) The accelerated durability test of Fe–ZIF’. l) Polarization curves and power density‐voltage plots of H_2_/O_2_ fuel cells using Fe–ZIF’ and Fe–N/CNT‐2. m) Accelerated stress tests of H_2_/O_2_ fuel cells using Fe–ZIF’ and Fe–N/CNT‐2 at 0.4 and 0.7 V. f–m) Reproduced with permission.^[^ ^86^ ^]^ Copyright 2019, Wiley‐VCH.

So far, most carbon matrixes of Fe–N–C catalyst have an insufficient level of graphitization, resulting in low corrosion resistance and inevitable active sites loss and the reduction of TOF.^[^ ^53^, ^82^ ^]^ Moreover, a carbon matrix with a low degree of graphitization is not conducive to electron transfer and conduction.^[^ ^43^ ^]^ In general, the porous carbon support has higher electrochemical surface area (ECSA) and specific surface area, but has poor electrochemical oxidation resistance.^[^ ^83^ ^]^ Graphitized carbon support is not easy to be electrochemically oxidized under high electrode potentials, and it can also effectively prevent the catalytic site from being submerged by water and reduce resistance loss caused by poor conductivity in the mass transfer process, but the dispersibility of metal atoms in it is poor.^[^ ^84^ ^]^ Therefore, it is a significant challenge to prepare a porous graphite carbon framework Fe–N–C catalyst with sufficient FeN*_x_* moieties to achieve efficient and stable ORR. Chen's group demonstrated a thin carbon sheets and foraminous carbon balls hybrid composite Fe–N–C catalyst (PANI–Fe–MCS) with remarkable electrocatalytic performance (**Figure** **10**a).^[^ ^85^ ^]^ Large electroactive surface area, excellent pores, and adjustable chemical structure are beneficial to the increased activity. In particular, the graphene layer and graphitized carbon support formed in situ have a high degree of graphitization, which can enhance the ability to resist carbon corrosion and effectively avoid the loss of active sites (Figure 10b,c). Both the long‐term stability test of the H_2_/O_2_ fuel cell with the PANI–Fe–MCS catalyst at a voltage of 0.40 V and the accelerated cycle test in 0.5 m H_2_SO_4_ saturated with O_2_ confirmed its great durability (Figure 10d,e). Kang et al.^[^ ^86^ ^]^ reported the carbon nanotubes incorporated with dense FeN*_x_* centers and surrounded by a graphitic nitrogen‐rich environment (Fe–N/CNT‐2), which showed high ORR catalytic activity and durability in fuel cells (Figure 10f). The adjacent graphite nitrogen realized a better filling of the *d*‐band orbital, and reduced on‐site magnetic moment (lowered spin) of Fe atom at the same time, thereby significantly enhancing the intrinsic activity. The higher graphitization degree of the carbon matrix made the catalyst have higher corrosion resistance (Figure 10g,h). In addition, the meso/macroporous carbon nanotube network was also conducive to water removal. Therefore, compared to the ZIF‐8‐derived Fe–N–C catalyst with low degree of graphitization (Fe–ZIF’), the stability curve of Fe–N/CNT‐2 in O_2_‐saturated 0.1 m HClO_4_ using RDE revealed a smaller performation attenuation (Figure 10i). Although the H_2_/O_2_ fuel cell with Fe–N/CNT‐2 had a voltage slightly lower than that of Fe–ZIF’ at high current densities (Figure 10l). During the long‐term polarization process at 0.40 V for 30 h, Fe–N/CNT‐2 showed better stability than Fe–ZIF’ (Figure 10m). The results of accelerated stress testing of these two catalysts also further confirmed the stability of the graphitized carbon material with high‐density active sites (Figure 10j,k).

#### 4.3.2 Edge Regulation

Edge defects are extremely common in carbon materials, and can usually be divided into armchair‐shaped and jagged edges. Compared to the base surface, the edge tends to possess special thermodynamic and electrochemical properties.^[^ ^7^, ^87^ ^]^ Generally, carbon oxidation is more likely to occur first at the defect‐rich edge.^[^ ^88^ ^]^ Therefore, electrocatalysts with exposed flat surfaces tend to be less prone to oxidation than those with exposed edges. Consequently, controlling the edge of the carbon matrix is also an effective means to slow down carbon corrosion. According to the reactivity, Baek et al. employed O_2_, H_2_O, CO_2_, and H_2_ to terminate the free edge of the graphite nanosheets by ex situ or in situ methods, and obtained samples with different edge oxidation groups (EOG) labeled EOG‐O_2_, EOG‐H_2_O, EOG‐CO_2_, EOG‐H_2_.^[^ ^89^ ^]^ The accelerated chemical corrosion test (ACCT) results for H_2_O_2_ and electrochemical stability test in the O_2_‐saturated 0.5 m H_2_SO_4_ solutions using RDE showed that the ether ring ((G)C–OC–C(G) in EOG‐O_2_) had the best stability to electrochemical and chemical oxidation. However, samples with abundant hydrogen groups such as (G)C–H, (G)C–OH, and (G)C–COOH were further oxidized and had poor electrochemical stability. Among them, the (G)C–COOH group was easily lost through gaseous carbon dioxide, suggesting the worst electrochemical/chemical stability. Besides, recently some researchers have proposed that reducing the content of the edge part of the catalyst structure is also an effective way to avoid corrosion of the carbon matrix.^[^ ^90^ ^]^

### 4.4 Rational Design of the Catalyst Layer

The activity and stability of the catalyst are closely related to its structure. Unlike Pt‐based catalysts, due to less active sites, to reach high catalytic performance, the Fe–N–C catalysts tend to have higher loading and thicker catalyst layers, which is not conducive to mass transfer.^[^ ^47^, ^55^ ^]^ Thus, a reasonable design of the catalyst layer structure is the sticking point to the replacement of commercial Pt/C with Fe–N–C catalyst. The key points of rational design of the catalyst layer are to improve mass transfer include adjusting the pore size distribution, hydrophobicity regulation, and increasing the density of active sites.

#### 4.4.1 Adjusting the Pore Size Distribution

In the catalyst layer, macropores and mesopores are conducive to mass transfer, while micropores are easier to support active sites.^[^ ^91^ ^]^ However, although more micropores are beneficial to the enhancement of catalytic activity, it is easy to form microporous flooding and filling, adversely affecting the stability.^[^ ^92^ ^]^ More importantly, compared to the active sites located in the micropores, the FeN*_x_* site located in the mesopores can be retained to the greatest extent after a long period of work.^[^ ^93^ ^]^ Generally, Fe–N–C electrocatalysts are prepared by pyrolyzing precursors rich in iron, nitrogen, and carbon, and the created FeN*_x_* sites are anchored in the carbon substrate. However, the pyrolysis process at high temperatures makes the obtained carbon skeleton rich in disordered micropores, causing the drastically dropped performance due to the flooding and filling of micropores. Therefore, reasonably designing the structure of the catalyst layer can allow it to own the reasonable pore size distribution, and intensive and highly exposed FeN*_x_* sites, effectively balancing the durability and activity of the catalyst. Mesoporous materials embrace large pore volume, tunable pore size, controllable geometry and high specific surface area, which enable them to increase the density of active sites and boost mass transfer, effectively avoiding performance degradation arising by microporous flooding and filling.^[^ ^94^ ^]^ The hard template method and soft template method are two common strategies for preparing hierarchically ordered mesoporous Fe–N–C materials.^[^ ^95^ ^]^

**Figure 11:**
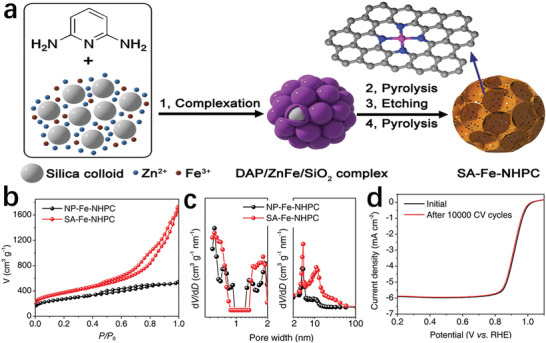
a) Schematic for the synthesis procedure of SA–Fe–NHPC. b) N_2_ adsorption/desorption isotherms of the SA–Fe–NHPC and other sample. c) The corresponding pore size distribution curves of the SA–Fe–NHPC and other sample. d) Linear sweep voltammetric curves before and after the accelerated durability test of SA–Fe–NHPC. a–d) Reproduced under the terms of the CC‐BY Creative Commons Attribution 4.0 International license (https://creativecommons.org/licenses/by/4.0).^[^ ^96^ ^]^ Copyright 2020, The Authors, published by Wiley‐VCH.

The hard template method could copy the rigid structure and specific morphology of the hard template into the new material, and the prepared product has good dispersibility and uniform pore size. Feng and co‐workerss used SiO_2_ as a hard template to synthesize a layered porous carbon material with dense and exposed FeN*_x_* sites (SA–Fe–NHPC), as shown in **Figure** **11**a.^[^ ^96^ ^]^ During the heat treatment of the 2,6‐diaminopyridine/ZnFe/SiO_2_ composite, zinc prevented the aggregation of iron atoms, and the SiO_2_ template promoted the formation of mesopores. Nitrogen adsorption–desorption analysis manifested that there were numerous mesopores in SA–Fe–NHPC, thereby significantly improving the accessibility of the FeN*_x_* part (Figure 11b,c). The SA–Fe–NHPC electrocatalyst showed superb ORR performance in a 0.1 m KOH aqueous solution saturated in O_2_, with a half‐wave potential (*E*_1/2_) of 0.93 V. After 10 000 cycles of cyclic voltammetry (CV) scanning, the *E*_1/2_ of the catalyst was reduced by only 1 mV, showing the excellent stability (Figure 11d).

**Figure 12:**
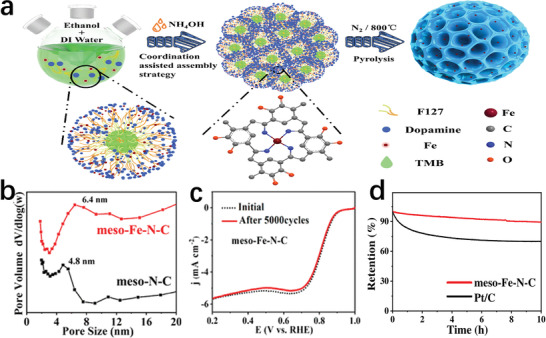
a) Schematic illustration of a formation process for the meso‐Fe–N–C. b) The pore size distribution curves of the meso‐Fe–N–C and meso‐N–C. c) The accelerated durability test of the meso‐Fe–N–C. d) The chronoamperometric responses of the meso‐Fe–N–C and Pt/C. a–d) Reproduced with permission.^[^ ^98^ ^]^ Copyright 2020, American Chemical Society.

However, the hard template method has a very low yield and its preparation process is complex, which makes it difficult to achieve mass production. The soft template method mainly forms clusters with certain structural characteristics through intermolecular or intramolecular interaction.^[^ ^97^ ^]^ The yield is often higher and the process is simpler, so it is more likely to be applied to industrial production. Zhou et al.^[^ ^98^ ^]^ employed Pluronic F127 as a soft template to prepare a Fe and N co‐doped mesoporous nanospheres (meso‐Fe–N–C) by a coordination‐assisted polymerization assembly strategy (**Figure** **12**a). The meso‐Fe–N–C had a monodisperse spherical shape, with an obvious ordered mesoscopic structure (Figure 12b). In a 0.1 m KOH solution saturated with O_2_ using RDE, there was no significant degradation of the catalyst after 5000 cycles (Figure 12c). The chronoamperometric responses test showed that meso‐Fe–N–C had much higher stability than Pt/C (Figure 12d). In addition, the half‐wave potential of this catalyst was also as high as 0.846 V. These test results evidenced that the mesoporous material prepared by this soft template method had high activity and remarkable stability.

#### 4.4.2 Hydrophobicity Regulation

**Figure 13:**
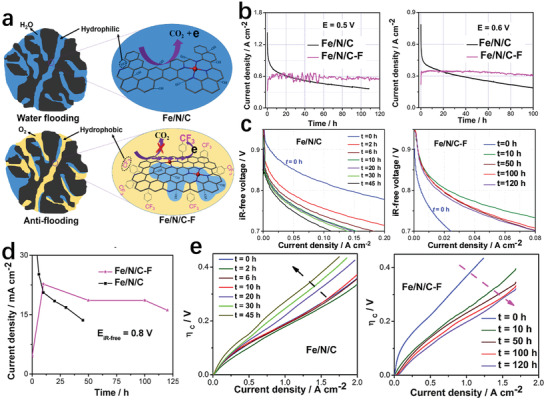
a) Schematic diagram of water flow in the micropores of Fe/N/C and Fe/N/C–F. b) Stability tests of H_2_/O_2_ PEMFC using the Fe/N/C–F and original Fe/N/C as cathodes at 0.5 and 0.6 V. c) Polarization curves of the H_2_/O_2_ fuel cells using two Fe–N–C catalysts before and after fluorination at different times after the start of the stability test at 0.5 V. d) The current density of the two catalysts at 0.8 V changes with time. e) The *η*_c_ curves of two Fe–N–C catalysts before and after fluorination at different times after the start of the stability test at 0.5 V. a–e) Reproduced with permission.^[^ ^99^ ^]^ Copyright 2017, Wiley‐VCH.

Another reason for the loss of catalyst performance due to the flooding of micropores is the poor hydrophobicity of the surface of the micropores.^[^ ^39a^ ^]^ Therefore, treating the surface of the catalyst to make it more hydrophobic is beneficial to prevent the micropores from flooding. Besides, water management in this way can also slow down possible iron demetalization and carbon corrosion enhanced by micropore flooding. Sun et al.^[^ ^99^ ^]^ proposed a method of treating Fe/N/C materials through surface fluorination to increase the hydrophobicity of the surface. The covalent grafting of hydrophobic trifluoromethylphenyl (Ar–CF_3_) on the Fe/N/C–F obtained by fluorination made the carbon substrate more hydrophobic and electron withdrawing (**Figure** **13**a). The hydrophobicity enhanced the ability of the catalyst to resist the micropores flooding. The reduction in water flow contact with the carbon substrate also reduced the occurrence of carbon corrosion. Moreover, the electron‐attracting carbon substrate after fluorination was not conducive to the progress of the carbon oxidation process. The long‐term potentiostatic test results of H_2_/O_2_ fuel cells with original Fe/N/C and Fe/N/C–F as cathodes at 0.5 and 0.6 V showed that the fluorination treatment greatly improved the stability of Fe/N/C catalyst (Figure 13b). The iR‐free polarization curves were used to characterize the intrinsic activity of Fe–N–C and Fe–N–C–F (Figure 13c). The current density corresponding to 0.8 V in Figure 13c was summarized as Figure 13d to estimate the stability of the catalyst. The performance of Fe–N–C decayed rapidly, while the activity of Fe–N–C–F decreased slowly after a rapid increase. The concentration overpotential (*η*_c_) of original Fe–N–C and Fe/N/C–F obtained by subtracting the cell voltage obtained by the Tafel equation from the iR‐corrected voltage could be used to characterize the mass transfer performance (Figure 13e). The original Fe–N–C catalyst has improved its mass transfer capacity due to the dissolution of Fe particles in the first 2 h. From 2 to 8 h, the mass transfer capacity did not change much, and the catalyst might reach an equilibrium state in which the dissolution of iron clusters improves the mass transfer capacity and carbon corrosion causes an increase in hydrophilicity. After working for 10 h, the equilibrium state was destroyed and carbon corrosion dominated, so the mass transfer capacity was greatly reduced. However, as the work progresses, the mass transfer capacity of Fe/N/C–F was steadily improved. The stable oxygen transport process of Fe/N/C–F was protected under the action of hydrophobic Ar–CF_3_. In the initial stage, the lack of water in the catalyst, as the most influential factor in the initial stage, was improved by the gentle oxidation of its surface. Therefore, the mass transfer capacity of the catalyst could be rapidly improved.

#### 4.4.3 Increasing the Density of Active Sites

**Figure 14:**
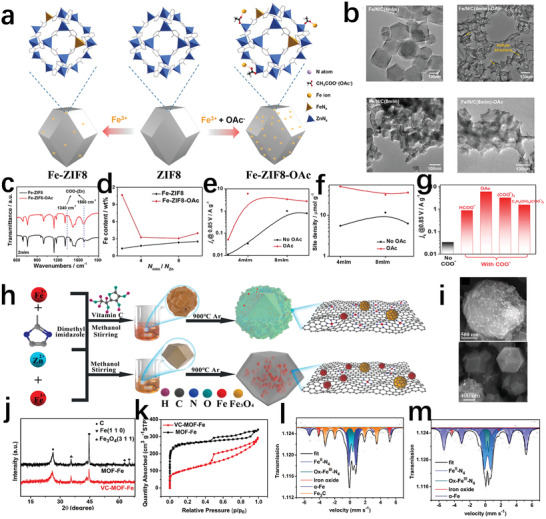
a) Schematic illustration of the synthesis of Fe–ZIF8 and Fe–ZIF8–OAc. b) Effect of OAc on the morphology of Fe–N–C catalyst. c) Fourier transform infrared spectra of Fe–ZIF8 and Fe–ZIF8–OAc. d) Fe content of Fe–ZIF8 and Fe–ZIF8–OAc at different molar ratios of 2‐methylimidazole ligand and Zn ion (*N*_mlm_/*N*_Zn_). e) Mass activity at 0.85 V (RHE) of Fe–ZIF8 and Fe–ZIF8–OAc at different *N*_mlm_/*N*_Zn_. f) Active sites density of Fe–N–C catalysts with and without OAc at different *N*_mlm_/*N*_Zn_. g) Mass activity at 0.85 V (RHE) of the five Fe–N–C catalysts assisted by four different carboxylates. a–g) Reproduced with permission.^[^ ^105^ ^]^ Copyright 2021, Wiley‐VCH. h) Schematic depicting the synthesis of VC–MOF–Fe and MOF–Fe. i) SEM image of VC–MOF–Fe and MOF–Fe. j) XRD patterns of Fe–N–C catalysts with and without vitamin C. k) N_2_ sorption isotherms of Fe–N–C catalysts. l) Fe Mössbauer spectra of VC–MOF–Fe. m) Fe Mössbauer spectra of MOF–Fe. h–m) Reproduced with permission.^[^ ^21a^ ^]^ Copyright 2021, Elsevier.

Increasing the density of the active site is very beneficial for optimizing the structure of the catalyst layer and avoiding mass transfer loss. In addition, the numerous active sites is beneficial to enhance the selectivity of the four‐electron ORR, which prevents hydrogen peroxide from damaging the catalyst structure.^[^ ^100^ ^]^ On the other hand, most ORR proceeds at the triple‐phase boundary (TPB) of catalyst–electrolyte–oxygen.^[^ ^101^ ^]^ The highly exposed TPB enhances the accessibility of active sites and effectively enlarges the part of active sites involved in the actual reaction.^[^ ^102^ ^]^ Besides, the highly exposed TPB facilitates mass transfer, thereby effectively reducing the impact caused by microporous flooding and filling and boosting the utilization of active sites.^[^ ^10c^ ^]^ Therefore, the construction of Fe–N–C structures with sufficiently exposed TPB and high‐density active sites is of great significance for optimizing the catalyst structure and increasing the catalyst mass activity.^[^ ^103^ ^]^ However, it is difficult to realize, because Fe tends to agglomerate during high‐temperature sintering.^[^ ^104^ ^]^ As shown in **Figure** **14**a, Sun's team proposed a carboxylate‐assisted strategy to increase the density of active sites.^[^ ^105^ ^]^ In the ordinary way, Fe doping into ZIF‐8 is achieved by replacing Zn^2+^ with Fe^3+^. After adding acetate (OAc), Fe^3+^ can also enter the ZIF‐8 structure in the form of Fe^3+^–OAC–Zn^2+^(Figure 14c). Moreover, the morphology of the Fe–ZIF8–OAc would change from a dodecahedron to a hollow structure after adding OAc (Figure 14b). This structure is more conducive to exposing TPB and increasing mass transfer. After adding OAc, the active center density and mass activity of such prepared Fe–N–C catalysts at different molar ratios of 2‐methylimidazole ligand and Zn ion (*N*_mlm_/*N*_Zn_) are significantly improved (Figure 14d–f). The results of using different forms of carboxylate to study the performance of the catalyst show that all ORR activities can increase by more than 10 times, which proves the high efficiency of this strategy (Figure 14g). Our group found that Vitamin C incorporated in ZIF‐8 can react with ferrous ions to form complexes and reduce the concentration of exposed metal ions on the surface of ZIF‐8, thus increasing the density of Fe–N_4_ sites with fewer Fe particles in VC–MOF–Fe (Figure 14h,j).^[^ ^21a^ ^]^ Fe Mössbauer spectrum of VC–MOF–Fe and MOF–Fe further evidenced that the density of Fe–N_4_ in VC–MOF–Fe increased significantly (Figure 14l,m). This confirmed the unique role of vitamin C in increasing active sites. Besides, it also revealed that vitamin C could corrode ZIF‐8, which can increase the contact area and promoted effective contact between reactants and active sites (Figure 14i,k).

## 5 Conclusions and Outlook

As a classic model of transition metal–heteroatoms–carbon (TM–H–C) catalyst, although the Fe–N–C catalysis system can effectively drive ORR, its poor durability/stability seriously confines their commercial applications. Thus, this review discusses the relationship between active sites and stability of Fe–N–C catalysts. N–C, Fe@N–C and FeO*_x_* (C*_y_*) are often not stable enough or have insufficient activity. FeN*_x_* C*_y_* and Fe_2_N*_x_* C*_y_* can achieve excellent balance of activity and stability. In addition, this article summarizes possible decay mechanisms, including microporous flooding, Fe demetallization, protonation of N‐groups, carbon corrosion, resistance loss, and micropore filling. Importantly, some effective strategies to reduce performance degradation of the catalyst, such as strengthening the electronic coupling, avoiding the attack of H_2_O_2_, increasing the oxidation resistance of carbon substrate, and rational design of the catalyst layer, are also analyzed and summarized. Although the stability improvement of Fe–N–C catalysts has achieved some results in the past few years, they are still not ideal for practical applications. Further improvement of Fe–N–C stability is still urgent. In future, for the challenge, some issues and perspectives are proposed as follows:

1. Although some strategies have effectively improved the stability of Fe–N–C catalysts to some extent, the loss of catalytic performance is still relatively large. Therefore, the activity and stability must be balanced to reach the standards of industrial applications.
2. Due to the complexity of the reaction process and intermediate products in the ORR catalyzed by Fe–N–C catalysts, the exact degradation mechanism is still unclear, hindering the improvement of Fe–N–C catalyst stability. Thus, the application of advanced characterization techniques such as in situ test is essential for in‐depth understanding of the catalyst decay process.
3. The stability test in the three electrode system is relatively limited to comprehend the degradation of catalysts. This is because in such a system, due to the existence of liquid electrolytes such as KOH and H_2_SO_4_, the OH^−^ or H^+^ is easy to transfer. By comparison, in the two‐electrode system, the proton transfer is extremely limited by the distribution and dosage of the solid electrolyte such as Nafion. Accordingly, it is necessary to simulate the real working environment of the catalyst in fuel cells.
4. Most stability improvement strategies are often aimed at one or two degradation reasons of catalysts, in future the complex degradation process of Fe–N–C catalysts under working conditions shall be considered.
5. In the past, a lot of efforts have been made to boost the catalytic performance of Fe–N–C catalysts, while ignoring the mass transfer problem caused by the high catalyst loading. Thus, an important aspect of future research on such catalysts is for the structure design of catalyst layers, including increasing the active site density to thin the catalyst layer, and adjusting the pore size distribution and the order of catalyst layers.
6. Unlike the ordinary electrochemical test in acidic or alkaline solutions as electrolytes, when ORR catalyst is applied to fuel cells, most of the active sites will not be able to effectively catalyze the reaction due to the inability to contact Nafion or oxygen, leading to the great reduction of the triple‐phase boundary (TPB). Therefore, catalyst layers with more highly exposed TPB will be more advantageous in actual work.
7. In most of the previous research on the ORR process, the DFT calculation only involved the adsorption capacity of the active site on O_2_ molecules and did not calculate the O—O band fracture barrier. This is not conducive to reasonable evaluation of the trend of hydrogen peroxide production which seriously threatens catalytic performance.
8. A proper Fe—N bond length is of great significance for preventing Fe atoms from aggregation and shedding to stabilize the active site. At present, there are few researches on the precise regulation of Fe—N bond length. Future research should focus on in‐depth investigation on the relationship between the Fe—N bond length and the active site stability, so as to achieve effective dispersion and anchoring of metal atoms.

In short, the future development direction of Fe–N–C catalysts as unique model of TM–H–C materials shall focus on the exploration of new and more stable catalysts through the combination of experimental results and DFT calculations, the exact mechanism of the performance degradation, the most effective improvement strategies of the stability, and practical evaluation in the two‐electrode system.

## Conflict of Interest

The authors declare no conflict of interest.
